# Laboratory Diagnosis of Malaria: Comparison of Manual and Automated Diagnostic Tests

**DOI:** 10.1155/2017/9286392

**Published:** 2017-04-05

**Authors:** Samina Naz Mukry, Madiha Saud, Gul Sufaida, Kashif Shaikh, Arshi Naz, Tahir Sultan Shamsi

**Affiliations:** ^1^Division of Immunology & Applied Microbiology, Department of Post Graduate Studies & Research, National Institute of Blood Diseases & Bone Marrow Transplantation, ST 2/A Block 17, Gulshan-e-Iqbal KDA Scheme 24, Karachi, Pakistan; ^2^National Institute of Blood Diseases & Bone Marrow Transplantation, ST 2/A Block 17, Gulshan-e-Iqbal KDA Scheme 24, Karachi, Pakistan

## Abstract

Malaria is the second most prevalent disease in Pakistan resulting in ~30,000 annual deaths. In endemic countries like Pakistan precise and timely diagnosis of malaria is imperative to overcome the associated risks of fatal outcomes. Malarial parasite was screened in 128 malaria suspected patients and 150 healthy controls, by species-specific PCR, microscopy of blood smears, hemoanalyzer Sysmex XE-2100, and rapid test devices (First Response Malaria® and ICT Malaria Combo®). The microscopy detected MP in 126 samples (parasite load/*µ*l 386–53712/*µ*l); 71.094% were infected with* Plasmodium vivax* and 14.844% with* P. falciparum* while 14.062% had mixed* P. vivax* and* P. falciparum* infection. The mean parasite load for* P. vivax* and* P. falciparum* was 14496/*µ*l and 24410/*µ*l, respectively. The abnormal scattergrams of DIFF, WBC/ Baso, IMI channel, and RET-EXT on Sysmex XE-2100 supported 99.2% parasite detection, whereas only 93% of confirmed malaria cases were detected by both rapid tests. About 127 samples were positive by PCR. Since Sysmex XE-2100 automatically detected the presence of malarial parasite with high sensitivity, it can be a good option for presumptive diagnosis in endemic areas. Microscopy remains the gold standard to confirm MP in suspected patients. Rapid diagnostic tests have acceptable sensitivity and specificity.

## 1. Background

Malaria is a protozoal disease. It is a parasitic infection of red blood cells. In humans it is generally caused by five different species of* Plasmodium,* namely,* P. vivax, P. falciparum, P. malariae, P. knowlesi,* and* P. ovale*. According to an estimate about 40% of the world population lives in high malaria zone [1]. Pakistan is a malaria endemic country and it is the second most prevalent disease in Pakistan. Major causative agents of malaria in Pakistan are* P. vivax* and* P. falciparum *with* P. vivax *being more common [2]. Malaria caused by* P. falciparum* is more severe and may often lead to cerebral malaria and death especially in children. Initially malaria due to* P. vivax* was generally considered as milder and manageable compared to* P. falciparum* infection, but recent global reports suggest that* P. vivax* malaria may cause complications leading to death. The global mortality rate for* P. vivax* is documented as 0.1–1.6% [3]. Hence, beside* P. falciparum* the* P. vivax* malaria should also be closely monitored to avoid complications and mortality. Thus, timely diagnosis of malaria in endemic areas is vital for early treatment and prevention of fatal outcomes in cases of* P. falciparum*,* P. vivax,* or mixed* P. falciparum* and* P. vivax* malaria.

Due to the limitation of local health service resources, imprecise clinical diagnosis remains the basis of therapeutic care for the majority of febrile patients in remote malaria endemic areas of Pakistan, where laboratory diagnostics is often out of reach. Diagnosis based on clinical features alone has very low specificity and results in overtreatment [4] and emergence of drug resistant strains. In order to avoid this, the WHO recommends confirmed diagnosis of all malaria suspected cases before giving treatment [5, 6]. In Pakistan laboratory diagnosis of malaria is indispensable to avoid misdiagnosis as per national guidelines. For precise malaria diagnosis, several diagnostic approaches are employed in labs including microscopy, immune-florescence technique, immune-chromatographic testing (ICT), PCR, and use of hematological analyzers [7–10].

The microscopic detection of malarial parasite is generally considered as a gold standard in malaria diagnosis due to low cost and accessibility. Although cheap, specific, and sensitive this procedure requires an expert microscopist and may become unreliable, time-consuming, and laborious at low parasite densities of <1000 parasites/*µ*l [11].

Malaria caused by* P*.* falciparum* may become complicated and fatal if misdiagnosed or left untreated. In order to detect low level parasitemia and also to detect mono- or coinfection of different parasite species immune-chromatographic/rapid diagnostic testing devices were developed. These devices based on parasite antigens or panspecific aldolases are simple and easy to use. The rapid diagnostic tests (RDTs) for malaria detection are usually based on principle of sandwich ELISA. These are inexpensive and easily available tests and require no prior training. Tests can be performed and results can be interpreted following manufacturer's instructions. Several reports on sensitivity and specificity of various commercial ICT devices are available [12, 13]. Devices like NOW Malaria® and ICT Malaria Combo can simultaneously detect histidine-rich protein 2 (HRP2) of* P. falciparum* and aldolase of all the* Plasmodium *species. Reports on poor specificity of these devices for few species like* P. ovale* led to developing devices based on detection of panspecific parasite lactate dehydrogenase (pLDH) enzyme [14]. OptiMal® is an ICT device of choice as it has a sensitivity of about 100% and is more specific 95% [15].

Automated malaria detection by hemoanalyzer is another approach to suspect malaria in febrile patients. Abnormalities in scattergrams of flow-cytometry-based hemoanalyzers like Sysmex XE-2100 and Cell Dyn have been reported as an aid in diagnosing malaria followed by microscopic confirmation [16].

Molecular diagnostic technique like PCR has an edge over the manual microscopy and serodiagnosis by RDTs. It is a reliable technique and can be used for malaria diagnosis. Beside genus specific PCR, species-specific multiplex and nested PCR have been developed for malarial parasite (MP) detection at a threshold of even 1 parasite/*μ*l [17]. The PCR can be used as an internal quality control rather than being used as part of routine diagnosis as it is expensive and time-taking and needs trained individuals [18].

According to the national statistical survey in 2007 malaria results in ~30,000 annual deaths in Pakistan [19]. The disease may be fatal especially in children and nonimmune individuals so high sensitivity of diagnosis in malaria endemic areas is particularly important. Misdiagnosis due to poor specificity of diagnostic modalities may be another issue leading to increased drug pressure causing antimalarial drug resistance. Aside from the vector control; the malaria-related morbidity and mortality may also be controlled by timely and accurate diagnosis of infection [20].

In this study performance of Sysmex XE-2100, ICT Malaria Combo, and First Response Malaria for early detection of MP was evaluated with microscopy and PCR as gold standard and internal quality control, respectively.

## 2. Materials and Methods

This cross sectional study was conducted at National Institute of Blood Diseases and Bone Marrow Transplantation (NIBD), Karachi, Pakistan. The patients and controls were recruited after approval by ethical review committee of NIBD. The study protocol adhered to the tenets of the Declaration of Helsinki.

### 2.1. Study Population

Blood specimens (6000 *µ*l) were collected in EDTA tubes from patients admitted at NIBD with clinical suspicion of malarial infection. Following national/WHO guidelines of precise diagnosis prior to treatment for malaria management 128 patients with clinical suspension of malaria were selected for this study. As a control group, sampling from 150 healthy individuals without clinical symptoms of malaria or any other infection or disease was also performed. The control group was confirmed as “malaria negative” by microscopy and RDT at the time of enrollment. The study was conducted over a period of about 9 months from October 2013 to July 2014. Signed informed consent and detailed questionnaire were obtained from the study population.

### 2.2. Laboratory Procedures

The complete blood count (CBC) data from Sysmex XE-2100 was recorded following standard machine operating protocol. The data was then analyzed and compared with morphological data to set standard design for automated malaria diagnosis. Sensitivity and specificity of this machine were also evaluated.

The microscopic examination of Giemsa/Leishman stained thick and thin blood smear for malaria diagnosis is the gold standard. Thick smear can detect parasite even in low densities since high volume of infected specimen is screened while thin smear helps in species differentiation. For microscopy, both thin and thick smears were prepared immediately in duplicate to avoid any discrepancies in morphological detection of malarial parasites in blood. The smears were stained with 4% Giemsa's/Leishman's stain and observed according to WHO standard guidelines by three independent observers.

The immunochromatographic testing was performed using two different RDTs, that is, ICT Malaria Combo and First Response Malaria, on fresh blood samples (not more than three hours old) as per supplier's instructions.

Molecular detection of malarial infection based on polymerase chain reaction (PCR) was performed using previously designed primers by Padley et al. 2003. Parasite DNA was extracted from fresh EDTA containing blood using QIAamp DNA Mini Kit (Qiagen, USA, Cat. number 51306). The extracted DNA was amplified and species were identified. The recorded results by agarose gel electrophoresis were then used as quality control to countercheck the data obtained by other diagnostic tests.

### 2.3. Data Analysis

The data was analyzed by SPSS version 17. The PL was calculated by multiplying number of asexual stages of parasite observed by microscopy with absolute RBCs count per 2500 RBCs. To assess sensitivity and specificity, results of microscopy, automated hemoanalyzer, and RDT were compared with PCR results. The sensitivity was calculated as the proportion of positive test results obtained among samples scored as containing malaria parasites by PCR; the specificity was the proportion of negative test results obtained among samples whose PCR results were negative. Positive and negative predictive values were also calculated as the proportion of true positive or true negative results among all samples scored as positive or negative by PCR, respectively. Youden's J-index was also calculated for comparative performance analysis of different tests.

## 3. Results

During the present study about 126 samples were found to be parasite positive by microscopy. Of these 126 samples, about 91 (71.094%) were infected with* Plasmodium vivax* and 19 (14.844%) with* P. falciparum* while mixed infection of* P. viva*x and* P. falciparum* was observed in 18 (14.062%) samples. Parasite load/*µ*l (PL) was also estimated by microscopy to evaluate the degree of severity of malaria. Only parasite load greater than 350 parasites/*µ*l was observed; none of the patients had very low parasitemia (Table 1). The mean parasite load for* P. vivax* and* P. falciparum* was 14496/*µ*l and 24410/*µ*l, respectively.

Like other diseases commercially prepared immune-chromatographic rapid diagnostic tests (RDTs) are available to detect malaria. The ICT Malaria Combo and First Response Malaria were compared for their efficacy. Among the two tested devices First Response Malaria seemed to be better with a sensitivity of 91.52% (95% CI: 87.52–95.52; Table 2). The positive predictive value of this device was 93.90% (95% CI: 91.10–96.70). The results are comparable with the gold standard microscopy. Based on their Youden's J-index of above 0.8 both the devices fall in category of very good diagnostic modalities.

**Figure 1:**
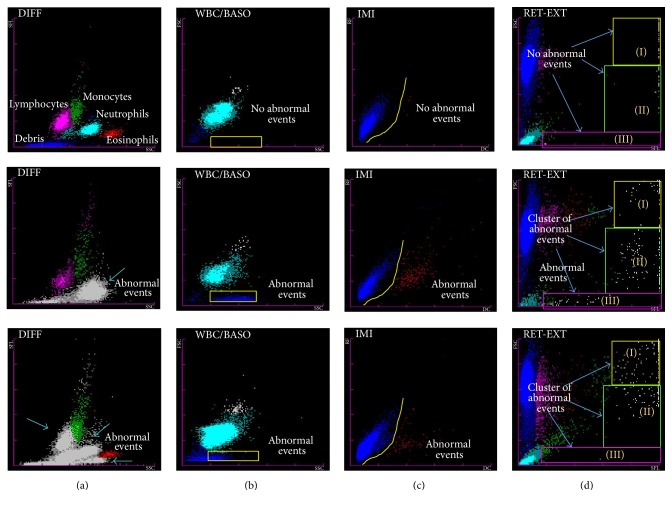
Parasitemia (*P. falciparum and P. vivax*) associated abnormalities in Sysmex XE2100 channels compared with normal sample at the top. (a) DIFF channel: the blue arrow indicates abnormal events depicting pseudoeosinophilia and graying of neutrophil cluster and double neutrophil and eosinophil populations; (b) WBC/BASO channel: yellow box shows parasite containing ghost RBCs; (c) IMI channel: comparatively more immature cells in area below yellow line in malaria positive cases; and (d) RET- EXT channel: presence of cluster of abnormal cells in any of the sectors extending vertically down and moving horizontally towards the *y*-axis.

**Figure 2:**
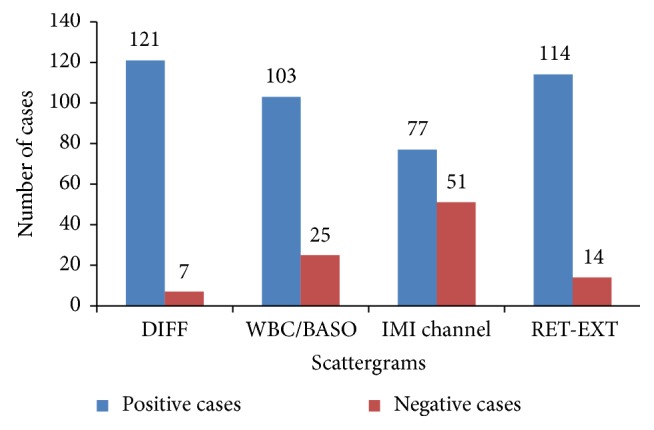
Abnormal scattergram in Sysmex XE2100 of malaria positive cases.

The Sysmex XE-2100 is generally used to record the routine hematological parameters as first-line screening test for any febrile patient. In the present study abnormal scattergrams on this analyzer were used for presumptive diagnosis of malaria. A total of about 126 cases were categorized as malaria suspected cases, on the basis of abnormal scattergrams in DIFF, WBC/Baso, IMI, and RET-EXT channels. Youden's J-index of automated hematological analyzer Sysmex XE-2100 for malaria detection was 0.98. The test seemed comparable with gold standard microscopy (Table 2). The pseudoeosinophilia and graying of neutrophil cluster and double neutrophil and eosinophil populations in DIFF channel were observed (Figure 1). In WBC/BASO channel, more than seven dots along the *x*-axis between first and third vertical marking were observed in case of* P. vivax* infection only. Increased signals (dots) in basophil region were also observable in approximately all cases of* P. vivax,* whereas there were no basophils in peripheral film. In case of malaria infection, multiple gray dots in the middle area were observed in the IMI channel despite the absence of immature granulocytes (myelo- and metamyelocyte) and any fluorescent signals above neutrophils in DIFF channel. Furthermore, the presence of gray dots along right side of the box extending vertically down and moving horizontally towards the *y*-axis in RET-EXT channel was indicative of* P. falciparum* infection (Figure 1). The abnormalities in DIFF channel were observed in 95% cases while the percentages of positive cases for WBC/Baso, IMI, and RET-EXT channels were 79%, 59%, and 83%, respectively. None of the samples from the control group showed abnormalities in any of these channels (Figure 2).

**Figure 3:**
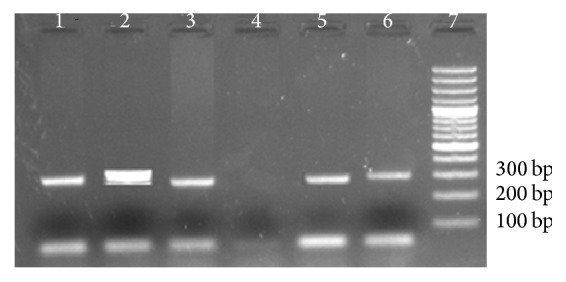
Agarose gel electrophoresis of amplified product obtained by 16S rRNA PCR using* Plasmodium* species-specific primers (Padley et al.). Lane 1:* P. vivax; *Lane 2: Mixed* P. vivax *+* P. falciparum; *Lane 3:* P. falciparum; *Lane 4: negative; Lane 5:* P. falciparum*; Lane 6:* P. vivax*; Lane 7: 100 bp ladder.

The species-specific PCR, being the internal quality control, was the most sensitive and specific test. It detected 127 malaria positive cases altogether (Table 2). None of the patients had infection caused by* P. malariae* and* P. ovale*. The product size for* P. vivax* and* P. falciparum* was 300 bp and 276 bp, respectively (Figure 3). The PCR also detected a case of* P. vivax* otherwise missed by microscopy. Furthermore, a case of* P. vivax* with low parasitemia (PL: 386/*µ*l) was missed by PCR. It can thereby be suggested that even PCR may overlook infection with low levels of parasite in blood. This sample was found to be positive by microscopy on third reexamination by expert microscopist.

On comparison the specificity and sensitivity of microscopy and automated hemoanalyzer were similar (Table 2). The species differentiation/identification by hemoanalyzer was not as obvious as with microscopy. So the results on hemoanalyzer may predict malaria but needs further confirmation by the gold standard microscopy. Hence, microscopy of thick and thin film remains the gold standard. Rapid diagnostic tests have acceptable sensitivity and specificity.

## 4. Discussion

The present study was designed to evaluate the utility in terms of efficiency of existing routine malaria diagnostic tests compared with gold standard microscopy and PCR as internal quality control. Microscopy being the gold standard was the only test producing quantitative results in the present study. The sensitivity of microscopy by thick smear is 5–10 parasites/*µ*l. It is cheaper when compared with other methods. The only limitation is the risk of human error and thus observer's expertise is required [21]. In the present study all the slides were observed by three microscopists independently but a clinically unapparent case of* P. vivax* malaria was missed. This is in line with other studies where cases were either missed or misdiagnosed by microscopy [22].

Two commercially prepared rapid test devices, that is, ICT Malaria Combo and First Response Malaria, were compared for their efficiency with microscopy as gold standard. Both the test devices were accurate with the accuracy of 93.16% (95% CI: 90.16–96.16) and 93.81% (95% CI: 89.7–97.7), respectively. The First Response Malaria was found to be more efficient device with a sensitivity of 91.52% (95% CI: 87.52–95.52). The sensitivity is comparable with an earlier study by Bharti et al. [23] on First Response Malaria (sensitivity: 93%). On the other hand the sensitivity for ICT Malaria Combo was found to be lower (90.83%; 95% CI: 86.83–94.83) than the reported value of 95.7% by Grobusch et al. [9] (Table 2).

Recent research is directed to detect malaria on the basis of the abnormal scattergrams of flow-cytometry-based automated hemoanalyzers [16, 24, 25]. First few reports were from Cell Dyn and Sysmex XE-2100. Extensive studies were done by Korean scientists explaining the utility of abnormal DIFF scattergrams in detecting malaria on Sysmex XE-2100. Huh et al. [26] reported unclassified spots extending from neutrophils towards eosinophil area, two eosinophil populations, two neutrophil populations, and overlapping of neutrophil and eosinophil populations as the most common abnormalities observed in WBC scattergrams on Sysmex XE-2100. The abnormalities may be caused by hemozoin containing particles interfering with the machine's WBC detection system resulting in abnormal counting of hemozoin containing neutrophils as well as due to their detection as eosinophils near the neutrophil cluster. Yoo et al., in 2010 [27], found 15.7% cases with abnormal WBC scattergram like two neutrophil and two eosinophil populations in assessment of 413 malaria cases.

About 95% malaria cases had abnormal WBC scattergram during the present study. Most common abnormality was found to be graying of eosinophil and neutrophil populations (53.12%). Other common abnormalities were overlapping of eosinophil and neutrophil populations (20.30%) and two eosinophil populations (32.80%).

A rightward shift of RBC ghost in WBC/BASO and DIFF scattergrams was also very commonly found in most malaria positive cases. This can be attributed to the presence of extracellular pigment and RBC lysis which are reflected in that area [28]. Pseudoeosinophilia by machine compared with manual differential count was observed in 6.25% cases which is comparatively lower than previous report of 39% of cases of pseudoeosinophilia [16, 29].

The nucleic acid based detection of malarial parasites by PCR is a more sensitive and specific approach than the gold standard microscopy. During this study the species-specific qualitative PCR detected* P. vivax* in one sample which was MP negative by microscopy. Additionally, this particular sample was also negative by both RDTs while clear abnormal signals suggesting presence of MP were observed in scattergrams on Sysmex XE-2100. This observation is in line with another study conducted in Pakistan where real-time PCR detected 3 samples missed by microscopy [30]. Thus, we recommend use of PCR for accurate diagnosis of malaria in public reference centers involved in WHO guided malaria control program in Pakistan. Coleman and colleagues conducted a detailed surveillance study in Thailand on comparison of PCR and microscopy for the detection of asymptomatic malaria in* P. falciparum*/*vivax* endemic area [31]. They suggested that PCR is a more precise and reproducible test for the MP species identification and detection but its performance decreased markedly at low parasite densities, that is, <500/*µ*l. The influence of low PL on performance of PCR was also recorded during the present study where MP positive case of* P. vivax* infection (PL: 386/*µ*l) was found to be false negative (Table 2).

Our study was time bound (9 months). Observer's training is required for identification of abnormal signals in scattergram. It must also be borne in mind that abnormal scattergrams may be observed in other conditions like dengue, basophilic stippling, thalassemia, and chronic myeloid leukemia. Observed scattergram abnormalities can only depict the presence of MP and cannot be used to differentiate between species of* Plasmodia.*

## 5. Conclusion

Thus, after comparison it can be concluded that the microscopy of thick and thin films remains the gold standard for malaria diagnosis despite chances of human error. The microscopy may be confirmed with PCR since the specificity and sensitivity for PCR are the highest. In remote endemic areas where microscopy due to absence of expert microscopists seems impossible, automated hemoanalyzers can serve as a useful adjunct to timely clinical diagnosis of malaria. The positive signals on hemoanalyzers need further confirmation by microscopy and PCR up to species level at the nearest reference lab to avoid unnecessary treatment, leading to development of drug resistant strains of MP. The comparative high cost of PCR limits its applicability in most diagnostic labs in developing countries, and we hereby recommend it to be added as a mandatory confirmatory test at least at all national reference labs.

## Figures and Tables

**Table 1:** Parasite load/*µ*l as estimated by microscopy.

Parasites/*µ*l	*P. falciparum*	*P. vivax*
<500	1	1
>500	3	25
>5000	23	67
>50,000	2	5

**Table 2:** Performance analysis of different tests with species-specific PCR as internal control.

Variables	ICT Malaria Combo	First Response Malaria	Microscopy	Automated hemoanalyzer (Sysmex XE-2100)	PCR
True positive	109	108	126	126	127
True negative	150	150	150	150	150
False positive	8	7	0	0	0
False negative	11	10	2	2	1
Negative Predictive Value (95% CI)	93.16 (90.16–96.16)	93.75 (90.85–96.65)	98.68 (96.31–99.60)	98.68 (96.31–99.60)	99.33 (99.24–99.42)
Positive predictive value (95% CI)	93.16 (90.16–96.16)	93.90 (91.10–96.70)	99.20 (98.20–100.20)	99.20 (98.20–100.20)	100
Sensitivity (95% CI)	90.83 (86.83–94.83)	91.52 (87.52–95.52)	98.41 (96.40–100.40)	98.41 (96.40–100.40)	99.21 (98.20–100.20)
Specificity (95% CI)	94.90 (92.30–97.50)	95.54 (93.14–97.94)	100	100	100
Accuracy (95% CI)	93.16 (90.16–96.16)	92.80 (89.7–97.7)	99.28 (97.00–101.00)	99.28 (97.00–101.00)	99.64 (99.57–99.71)
Youden's J- index	0.86	0.87	0.98	0.98	0.99

## Abbreviations

MP: Malarial parasite
PL: Parasite load
CBC: Complete blood count
RDTs: Rapid diagnostic tests
HRP2: Histidine-rich protein 2.
